# Diurnal dynamic behavior of microglia in response to infected bacteria through the UDP-P2Y_6_ receptor system

**DOI:** 10.1038/srep30006

**Published:** 2016-07-21

**Authors:** Fumiko Takayama, Yoshinori Hayashi, Zhou Wu, Yicong Liu, Hiroshi Nakanishi

**Affiliations:** 1 Department of Aging Science and Pharmacology, Faculty of Dental Science, Kyushu University, Fukuoka 812-8582, Japan; 2 JSPS Research Fellow, 5-3-1 Kojimachi, Chiyoda-ku, Tokyo 102-0083, Japan; 3 OBT Research Center, Faculty of Dental Science, Kyushu University, Fukuoka, 812-8582, Japan

## Abstract

It has long been believed that microglia morphologically transform into the activated state by retracting their long processes and consuming pathogens when bacteria infect into the brain parenchyma. In the present study, however, we showed for the first time that murine cortical microglia extend their processes towards focally injected *Porphyromonas gingivalis*. This *P. gingivalis*-induced microglial process extension was significantly increased during the light (sleeping) phase than the dark (waking) phase. In contrast, focally injected ATP-induced microglial process extension was significantly increased during the dark phase than the light phase. Furthermore, in contrast to the P2Y_12_ receptor-mediated mechanism of ATP-induced microglial process extension, the *P. gingivalis*-mediated microglial process extension was mediated by P2Y_6_ receptors. The infection of bacteria such as *P. gingivalis* to the brain parenchyma may induce the secretion of UDP from microglia at the site of infection, which in turn induces the process extension of the neighboring microglia.

An etiological hypothesis suggests that periodontitis may be associated with cognitive decline in patients with Alzheimer’s disease (AD)^1^,^2^,^3^. *Porphyromonas gingivalis* and *Treponema denticola*, which are categorized as part of the red complex due to their association with severe forms of periodontitis, are invasive and virulent within their original niche where they induce gingival inflammation that leads to connective tissue degradation and alveolar bone resorption around the teeth^4^,^5^. Once the junctional epithelium that links the gingiva to the tooth enamel transforms to pocket epithelium, pathogenic bacteria induce bacteremia and initiate chronic low-grade systemic inflammation by infiltrating the local blood vessels^6^,^7^. Despite a clear cause and effect relationship between periodontitis and cognitive decline in AD has been demonstrated^8^,^9^, the precise mechanism underlying the relationship still remains unclear.

There is increasing experimental evidence that chronic systemic inflammation, due to chronic periodontitis, exerts detrimental effects on the brain function, including impairment of memory, through neuroinflammation. Two mechanisms have been proposed by which chronic periodontitis may lead to the progression of AD.

First, AD may be exacerbated by increased levels of peripheral proinflammatory molecules due to chronic low-grade systemic inflammation associated with bacterial infection^10^,^11^. The level of proinflammatory molecules in the brain increase with age, leading to enhanced sickness behavior induced by lipopolysaccharide (LPS), suggesting that aging microglia are over-responsive^12^,^13^. Some reports have suggested that the leptomeninges covering the surface of the brain parenchyma can transmit signals from systemic immune cells into the brain-resident microglia^14^,^15^. Notably, even microglia in middle-aged animals respond in an exaggerated manner to chronic systemic inflammation^3^,^16^.

Second, AD may be exacerbated by the direct infiltration of microbes and their virulent factors into the brain parenchyma. Using molecular profiling methodologies, two different studies identified seven *Treponema* species^17^ and LPS from *P. gingivalis*^18^ in AD brain tissue specimens. More recently, fungal materials, including materials from *Candida*, have been detected intra- and extracellularly in neurons from AD patients^19^. In addition to oral pathogens, viruses such as Herpes simplex virus Type 1^20^ and a number of bacterial infections, including *Chlamydophila pneumoniae*^21^ and *Borrelia burgdorferi*^22^, have been detected in the brains of AD patients. After infiltration in the brain, microbes and their virulent factors may directly induce neuroinflammation through activation of the brain-resident microglia. However, little information is available regarding the mechanism by which microglia detect and respond to infiltrated microbes and their virulent factors.

We recently provided the first evidence that murine cortical microglia contain PER-based circadian clocks^23^. However, the potential differences in the microglial reactions to brain damages or bacterial infection between the light (sleeping) phase and the dark (waking) phase are poorly understood. In the present study, we conducted two-photon laser microscopic analyses of microglial process motility to examine the potential diurnal variations in the dynamic behavior of the microglial processes in mice. We herein show the P2Y_6_ receptor (P2Y_6_R)-dependent diurnal microglial process extension in response to focally injected bacteria including *P. gingivalis*, which may provoke neuroinflammation and induce cognitive impairment.

## Results

### Diurnal variation in the morphological and dynamic behavior of microglial processes

**Figure 1:**
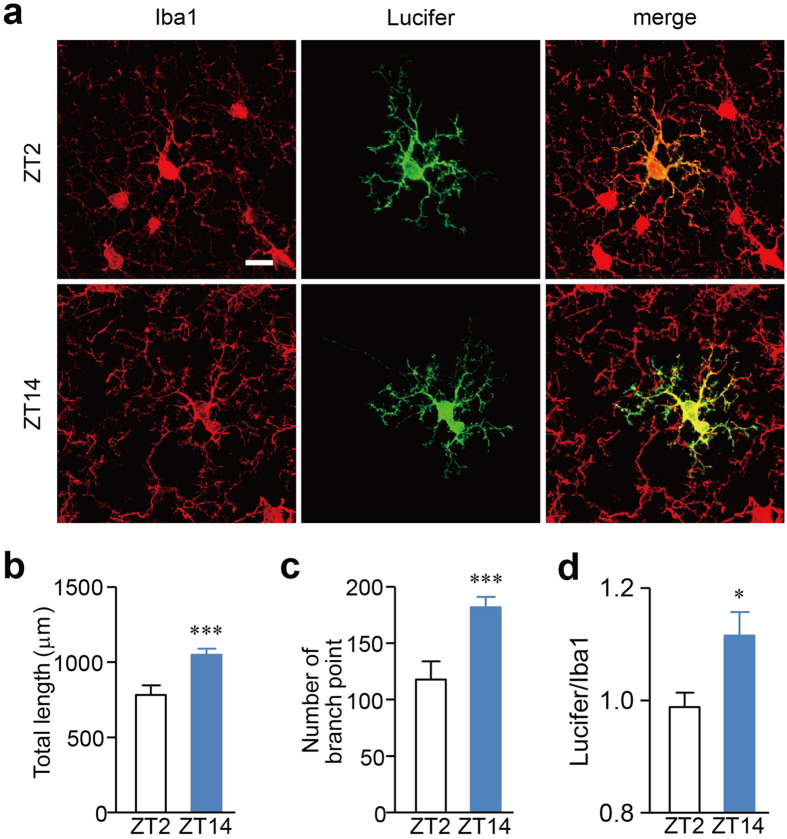
The diurnal variation in the morphological and dynamic behavior of the microglial processes. (**a**) The diurnal morphological variation in the intracellularly labeled cortical microglia. The Iba1-stained microglia (Iba1), intracellularly labeled microglia (lucifer) and their merged CLMS images. Scale bar: 10 μm. (**b,c**) The mean total length of the processes (**b**) and branch points (**c**) of the intracellularly labeled microglia at ZT2 and ZT14. The data are presented as the mean ± S.E.M. (N = 3 mice, n = 12–13 microglia each). A two-tailed unpaired *t*-test; ***p = 0.0005 (**b**), ***p = 0.0005 (**c**,**d**) The mean ratio of the total microglial process length of Iba1-immunostaind microglia to the intracellularly labeled microglia. The data are presented as the mean ± S.E.M. (N = 3 mice, n = 5 microglia each). A two-tailed unpaired *t*-test; *p* = 0.0325.

Our detailed morphological analyses showed that the mean total length and the mean number of branch points of Iba1-immunostained cortical microglia were significantly greater in the dark (waking) phase than the light (sleeping) phase^24^. However, we cannot rule out the potential underestimation of the total length of microglial processes due to the possible lack of antigenicity in the thin processes. To address this issue, we conducted a morphological analysis of microglia using the intracellular labeling method in fixed brain slice preparations. The Iba1-imunostained brain slices were further labeled intracellularly using Lucifer yellow (Fig. 1a). The mean total length and the mean number of branch points were significantly greater at ZT14 than at ZT2 (Fig. 1b,c). Although the mean total length measured in the intracellularly labeled microglia was significantly larger than that of the Iba1- immunostained microglia at ZT14 (Fig. 1d), the diurnal variation in the microglial morphology was consistent with our previous findings obtained in the Iba1-immunostained microglia.

### Differential diurnal variation in the dynamic behavior of the microglial processes in response to the focal injection of ATP and bacteria

**Figure 2:**
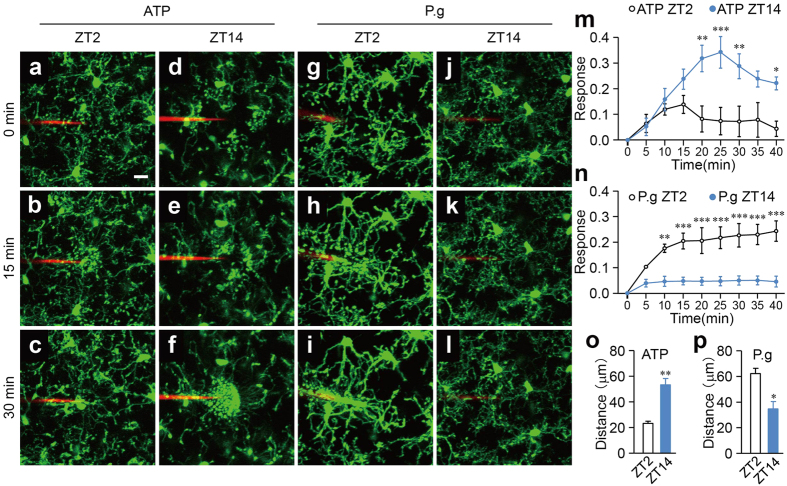
The differential diurnal variation in the dynamic behavior of the microglial processes in response to the focal injection of ATP and *P. gingivalis.* (**a–l**) The dynamic response of the microglial processes to the local injection of 10 mM ATP (**a–f**) and 3.6 × 10^5^ CFU ml^−1^ *P. gingivalis* (**g–l**) in an open-skull preparation of *CX3CR1*^*GFP*/+^ mice at ZT2 and ZT14. Scale bar: 10 μm. (**m,n**) The kinetics of the mean fluorescent change in the microglial response to ATP (**m**) and *P. gingivalis* (**n**) at ZT2 and ZT14. The data are presented as the mean ± S.E.M. (N = 3 mice, n = 3–6 each). A two-way repeated measure ANOVA with Sidak’s test (ZT2 versus ZT14); from 10 to 40 min: *p* = 0.9985, *p* = 0.6209, *p* = 0.0030, *p* = 0.0006, *p* = 0.0082, *p* = 0.0939, *p* = 0.0455 (**m**), *p* = 0.0030, *p* = 0.0002, *p* = 0.0001; from 25 to 40 min: *p* = 0.0001 (**n**–**p**) The maximum distance from the reactive microglia to the injection of ATP (**o**) and *P. gingivalis* (P.g) (**p**) at ZT2 and ZT14. The data are presented as the mean ± S.E.M. A two-tailed unpaired *t*-test; **p = 0.0042 (**o**), *p = 0.0173 (**p**).

We next examined the possible diurnal variations in the microglial response to neuronal damage. The mean motility speed of the individual processes following the focal injection of ATP was measured using a two-photon *in vivo* imaging system, since ATP release as a result of neuronal damage is important in mediating the microglial response^25^. Microglia extended their processes to the site of ATP injection at both ZT2 (Fig. 2a–c, Supplementary Video S1) and ZT14 (Fig. 2d–f, Supplementary Video S2). However, the mean microglial response was significantly greater at ZT14 than at ZT2 (Fig. 2m,o).

**Figure 3:**
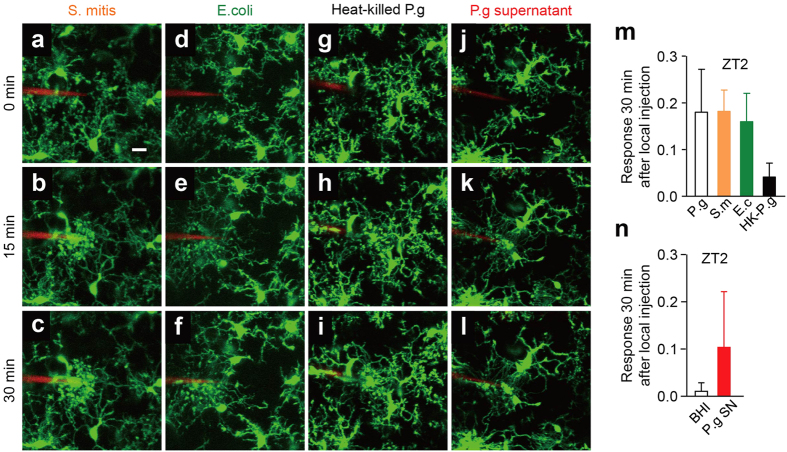
The effects of *S. mitis*, *E. coli*, heat-killed *P. gingivalis* and the supernatant prepared from *P. gingivalis* cultures on the microglial response. (**a–l**) The dynamic response of the microglial processes to the local injection of various bacteria (3.6 × 10^5^ CFU ml^−1^) at ZT2. (**a–c**) S. mitis (S.m), (**d–f**) E. coli (E.c), (**g–i**) heat killed P.g (HK-P.g; 95 °C, 10 min), (**j–l**) P.g culture supernatant (P.g SN). Scale bar: 10 μm. (**m**) The kinetics of the mean fluorescent change in the microglial response measured at 30 min after the injection of various bacteria. The data are presented as the mean ± S.E.M. (N = 3 mice, n = 4–5 each). A one-way ANOVA with a post hoc Tukey’s test; P.g vs S.m: *p* > 0.9999, Pg. vs. E.c: *p* = 0.9953, P.g vs. HK-P.g: *p* = 0.4244, S.m vs. E.c: *p* = 0.9939, S.m vs. HK-P.g: *p* = 0.4126, E.c vs. HK-P.g: *p* = 0.5521. (**n**) The kinetics of the mean fluorescent change in the microglial response measured at 30 min after the injection of *P. gingivalis*. The data are presented as the mean ± S.E.M. (N = 3 mice, n = 5–6 each). A two-tailed unpaired *t*-test; P.g vs BHI (P.g culture medium, as a control): *p* = 0.0856.

The cortical microglial response to the infected bacteria was also examined. We first examined the microglial response to the infected *P. gingivalis*, a major periodontal pathogen. Rather surprisingly, microglia also extended their processes to the site of *P. gingivalis* injection at both ZT2 (Fig. 2g–i, Supplementary Video S3) and ZT14 (Fig. 2j–l, Supplementary Video S4). In contrast to ATP, the mean *P. gingivalis*-induced microglial process extension was significantly greater at ZT2 than at ZT14 (Fig. 2n,p). It was also noted that the mean mRNA levels of proinflammatory-related molecules, including interleukin-6 (*Il6*), tumor necrosis factor-α (*Tnf*) and inducible nitric oxide synthase (*Nos2*), in MG6 microglial cells were significantly increased after infection with *P. gingivalis* without change in the cell viability (Supplementary Fig. S1a–e). We further examined the effects of *S. mitis*, a Gram-positive oral bacteria abundant in the oral cavity, and *E. coli* on the microglial process extension using the same two-photon *in vivo* imaging system as above. The focally injected *S. mitis* (Fig. 3a–c) and *E. coli* (Fig. 3d–f) also induced microglial process extension to a similar extent as *P. gingivalis* at ZT2 (Fig. 3m). These observations are consistent with the notion that the defense response to a bacterial infection has a circadian rhythm^26^,^27^.

To clarify the mechanism underlying the *P. gingivalis*-induced microglial process extension, the effects of heat-killed *P. gingivalis* and the supernatant prepared from *P. gingivalis* culture were examined. The heat-killed *P. gingivalis* was also able to induce the microglial process extension (Fig. 3g–i,m). In contrast, however, the supernatant prepared from *P. gingivalis* culture showed considerable variety in the induced microglial response (Fig. 3j–l,n) and the kinetics of the mean fluorescence of the microglial response failed to reach statistical significance compared to the control. Furthermore, focal injection of both *P. gingivalis* LPS and *N*-formylmethionine- leucine-phenylalanine (fMLP), a well-known chemotactic factor secreted by bacteria, failed to induce the microglial process extension (Supplementary Fig. S2a–j).

### The possible involvement of the UDP-P2Y_6_R system in the bacteria-induced microglial process extension

**Figure 4:**
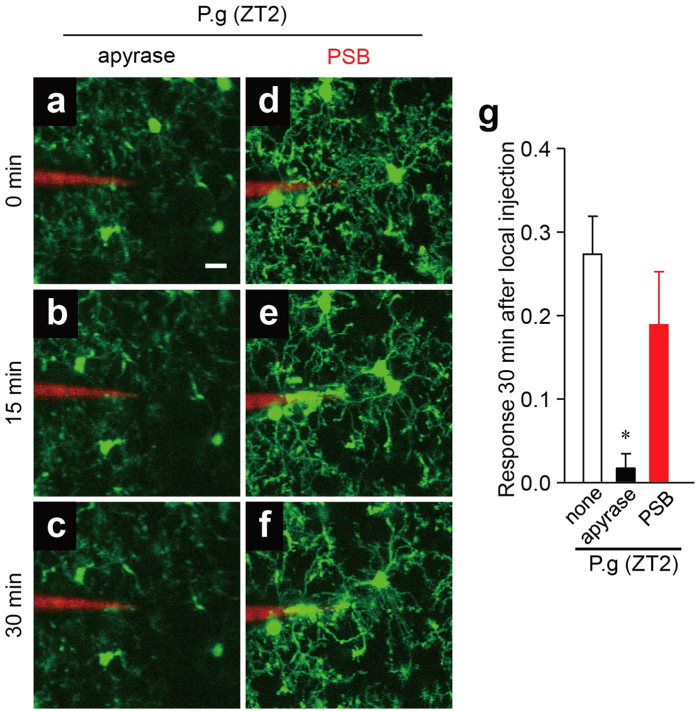
The possible involvement of extracellular nucleotides in the microglial response to bacterial infection. (**a–i**) The dynamic response of microglial processes to the local injection of *P. gingivalis* in the presence of various compounds at ZT2. (**a–c**) 5 U ml^−1^ apyrase, (**d–f**) 1 μM PSB. Scale bar: 10 μm. (**g**) The kinetics of the mean fluorescent change in the microglial response measured at 30 min after the injection of *P. gingivalis* in the presence of various compounds at ZT2. The data are presented as the mean ± S.E.M. (N = 3 mice, n = 3–4 each). A one-way ANOVA with Dunnett’s test; P.g vs. apyrase: *p = 0.0230; P.g vs. PSB: *p* = 0.4400.

We next examined the effects of apyrase, an enzyme which hydrolyzes extracellular nucleotides (UTP, UDP, ATP and ADP), and PSB, a selective inhibitor of P2Y_12_ receptor (P2Y_12_R), on the bacteria-induced microglial process extension. The extension at ZT2 was completely suppressed by apyrase (Fig. 4a–c,g) but not by PSB (Fig. 4d–g). ATP was not detected in the supernatant prepared from cultures of *P. gingivalis*, *S. mitis*, or *E. coli*, but it was detected in the supernatant prepared from the infection of MG6 microglial cells with bacteria, including *P. gingivalis*, *S. mitis*, and *E. coli* (Supplementary Fig. S3). However, ATP at a picomolar concentration cannot induce the microglial process extension. These observations suggest that bacteria focally injected into the somatosensory cortex provokes the focal secretion or leakage of extracellular nucleotides other than ATP, which in turn induces the microglial process extension.

**Figure 5:**
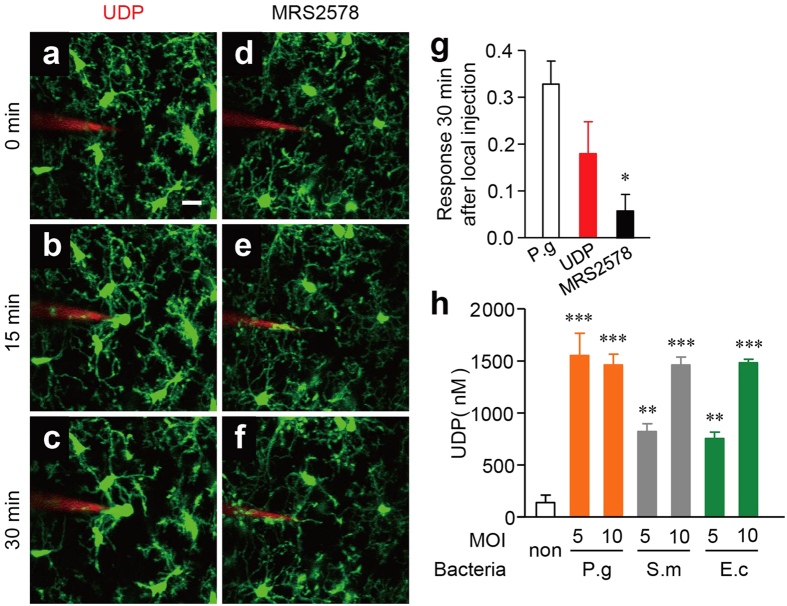
The possible involvement of UDP in the microglial response to bacterial infection. (**a–c**) The dynamic response of the microglial processes to the local injection of 10 mM UDP and *P. gingivalis* in the presence of 1 μM MRS2578 (**d–f**) at ZT2. Scale bar: 10 μm. (**g**) The kinetics of the mean fluorescent change in the microglial response measured at the 30 min after the injection of UDP and *P. gingivalis* in the presence of MRS2578. The data are presented as the mean ± S.E.M. (N = 3 mice, n = 5–6 each). A one-way ANOVA with Dunnett’s test; P.g vs. UDP: *p* = 0.2162, P.g vs. MRS3578: *p = 0.0277. (**h**) MG6 microglial cells released UDP after infection with bacteria at indicated MOI. P.g, *P. gingivalis*; S.m, *S. mitis*; E.c, *E. coli*. The data are presented as the mean ± S.E.M. (n = 4 each). A one-way ANOVA with Dunnett’s test as compared with the control; P.g 1:5, *p* = 0.0001; P.g 1:10, *p* = 0.0001; S.m 1:5, *p* = 0.0021; S.m 1:10, *p* = 0.0001; E.c 1:5, *p* = 0.0049; E.c 1:10, *p* = 0.0001.

We next investigated the potential involvement of the UDP-P2Y_6_R system in the bacteria-induced microglial process extension, as microglia express the metabotropic P2Y_6_R, whose activation by the endogenous agonist UDP triggers microglial phagocytosis^28^. A focal injection of UDP strongly induced the microglial process extension (Fig. 5a–c,g) and MRS2578, a specific inhibitor of P2Y_6_R, significantly inhibited the *P. gingivalis*-induced microglial process extension (Fig. 5d–g). As expected, UDP was not detected in the supernatant prepared from *P. gingivalis*, *S. mitis*, or *E. coli* cultures. However, UDP at micromolar concentration was detected in the supernatant prepared from the infected MG6 microglial cells with bacteria (Fig. 5h).

**Figure 6:**
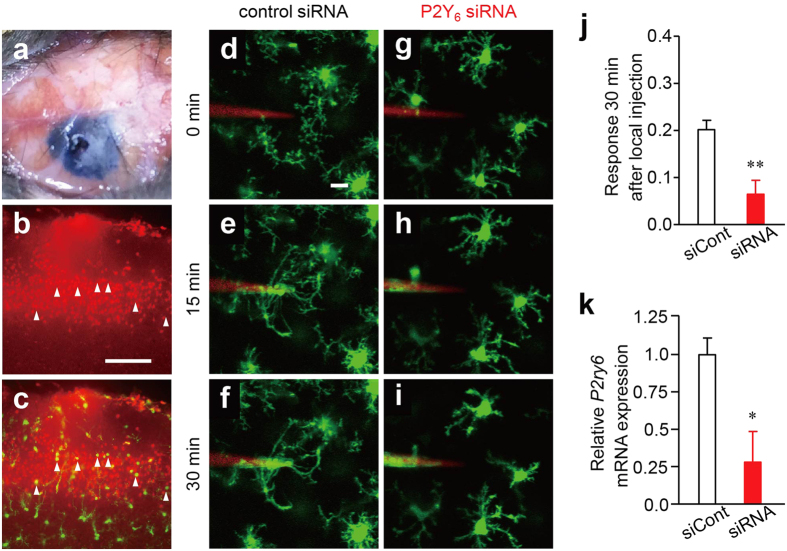
P2Y_6_R knockdown inhibited the dynamic behavior of the microglial processes in response to bacterial injection. (**a–c**) The determination of the transfection efficiency using BLOCK-iT Alexa Fluor Red Fluorescent Control. (**a**) The BLOCK-iT Alexa Fluor Red Fluorescent Control (20 pmol) was applied through a small craniotomy. (**b**) CLSM images showed the intracellular uptake of the BLOCK-iT Fluorescent Oligo (Red) in the somatosensory cortex at 24 h after transfection. (**c**) CLSM images merged with the BLOCK-iT Fluorescent Oligo (red) and microglia (green) in the somatosensory cortex of *CX3CR1*^+/*GFP*^ mice at 24 h after transfection. Scale bar: 10 μm. (**d–i**) The dynamic response of the microglial processes to the local injection of *P. gingivalis* after the administration of the control siRNA (**d–f**) and P2Y_6_R siRNA. Scale bar: 10 μm. (**j**) The kinetics of the mean fluorescent change in the microglial response measured at 30 min after the injection of *P. gingivalis* following the administration of the control siRNA (siCont) and P2Y_6_R siRNA (siRNA). The data are presented as the mean ± S.E.M. (N = 3 mice, n = 5 each). A two-tailed unpaired *t*-test; **p = 0.0051. (**k**) The relative *P2ry6* mRNA expression level after the administration of the control siRNA and P2Y_6_R siRNA. The data are presented as the mean ± S.E.M. (N = 3 animal, n = 14 each). A two-tailed unpaired *t*-test; *p = 0.0373.

We then evaluated the effect of *in vivo* gene silencing of P2Y_6_R using a small interfering RNA (siRNA) on the extension response. Figure 6a shows the diffusion area of the focally injected solution containing Evans blue on the surface of the somatosensory cortex. To optimize the conditions for gene silencing by siRNA, we first examined the transfection efficiency into microglia using BLOCK-iT Alexa Fluor Red Fluorescent Control (Thermo Fisher Scientific, Waltham, MA, USA). At 24 h after reverse transfection, the GFP-positive microglia in the cortical layers of 2–3 *CX3CR1*^+/*GFP*^ mice corresponded well with the BLOCK-iT Alexa Fluor Red-positive cells (Fig. 6b,c, arrowheads). #3 P2Y_6_R siRNA could deplete the *P2ry6* mRNA in the somatosensory cortex around the siRNA injection area (Fig. 6k). Based on the result, we utilized #3 siRNA in the subsequent *in vivo* imaging analysis of the microglial response. The gene silencing of P2Y_6_R significantly inhibited the *P. gingivalis*-induced microglial process extension (Fig. 6d–j). We then examined whether or not the cortical microglia regulated *P2ry6* gene expression, as these microglia were found to exhibit a circadian oscillation of *P2ry12* transcripts with a peak at ZT14^24^. The cortical microglia isolated using CD11b microbeads at 4 h-intervals over a 24-h period were found to exhibit a circadian oscillation of *P2ry6* transcripts with a peak at around ZT2 (Supplementary Fig. S4). The mean expression of *P2ry6* transcripts at ZT10 was significantly greater than at ZT14 (*p* = 0.0039). Taken together, these findings suggest that the UDP-P2Y_6_R system is the main underlying mechanism of the *P. gingivalis*-induced microglial process extension in the somatosensory cortex.

## Discussion

We herein show that the intracellularly labeled cortical microglia exhibit a diurnal variation in the morphology of their processes. Both the mean total length and number of branch points were significantly greater at ZT14 than at ZT2. In addition to our observations regarding the static morphology, we also noted diurnal variation in the dynamic behaviors of the microglial processes. The mean cortical microglial response to focally injected ATP was significantly greater at ZT14 than at ZT2. The ATP-induced microglial process extension is well known to be mediated by P2Y_12_R^25^. The E-box site (CACGTG), the target of the circadian clock effectors BMAL1 and CLOCK, is located 382 bp upstream of *P2ry12*. Using a luciferase reporter assay system targeting this region, we previously found that the diurnal changes in the *P2ry12* gene expression are regulated by the PER-based circadian clock of the cortical microglia^23^,^24^. The expression *P2ry12* peaking at ZT14^23^,^24^ may thus explain the exaggerated microglial response to the focal injection of ATP at ZT14.

Our observations here further showed that a focal injection of live bacteria, including *P. gingivalis*, *S. mitis*, and *E. coli*, into the somatosensory cortex strongly induced the microglial process extension. Furthermore, the functional blockade of P2Y_6_ R using a pharmacological inhibitor (MRS2578) or genetic depletion significantly attenuated the *P. gingivalis*-induced cortical microglial process extension. The focal injection of UDP also induced the microglial process extension. Therefore, injected bacteria, including *P. gingivalis*, *S. mitis* and *E. coli* may induce the secretion of UDP from the surrounding microglia at the injected site, thereby promoting the process extension of the neighboring microglia as well (Supplementary Fig. S5), since micromolar concentrations of UDP were detected in the supernatant prepared from the microglia infected with bacteria, but not in the supernatant of the bacterial culture alone. Microglia localized at the site of the bacterial injection are likely directly induced to secrete UDP by the injected bacteria. Further studies will be needed to clarify the mechanism underlying this bacteria-induced secretion of UDP from microglia.

There is increasing evidence that clock genes regulate the functions of peripheral immune cells^29^. A previous study found that the phagocytic activity of peritoneal macrophages exhibited circadian variation that peaked during the light phase and remained high through the dark phase^30^, enhancing the clearance of bacteria and dead cells at the beginning of the active phase. The expression of proinflammatory cytokines and chemokines in peritoneal macrophages, including IL-6, IL-12 and chemokine (C-C motif) ligand 5, was significantly greater when mice were challenged with systemic LPS during the active phase than during the inactive phase, suggesting a circadian gating of the endotoxin response^31^. More recently, the expression of IL-6 in brain-resident microglia has been found to peak at the beginning of the active phase^32^,^33^, and in the present study, the expression of P2ry6 also peaked at the beginning of the active phase (ZT10). The expression of Rev-erbα, another circadian effector, in the cortical microglia peaked at ZT18^23^. Furthermore, the promoter region of *P2ry6* does not contain the E-box site, but does contain the RORE site, the target of Rev-erbα, at 1700 bp upstream. These observations suggest that Rev-erbα suppresses the expression of *P2ry6* as well as IL-6 during the active phase. The expression peak of *P2ry6* may explain the elevated microglial response to the focal injection of *P. gingivalis* at ZT2. Therefore, the brain-resident microglia may enhance the clearance of bacteria infecting in the brain before starting the active phase, as the UDP-P2Y_6_R system is involved in the microglial process extension and phagocytic activity^28^. This finding is consistent with a previous report that the UDP-P2Y_6_R system enhances the clearance of invading *E. coli* by recruiting monocytes/macrophages^34^.

In conclusion, the intrinsic microglial molecular clock drives the peak expression of *P2ry12* and *P2ry6* at ZT14 and ZT2 in cortical microglia, respectively. Moreover, *P. gingivalis* was found to activate microglia to produce proinflammatory-related molecules (see Supplementary Figure S1). Therefore, it is reasonable to consider that microglial molecular clock could limit the over-reaction and inflammatory response of microglia in response to infected bacteria during active phase by circadian regulation of the *P2ry6* expression. Therefore, microglia may overreact to infected bacteria provoking excessive neuroinflammation in Alzheimer’s disease, because there is evidence for circadian rhythm disturbance and clock gene dysfunction in Alzheimer’s disease^35^.

## Methods

### Animals

*CX3CR1*^+/*GFP*^ mice on a C57/BL6J background (8–10 weeks old, Jackson Laboratory, Bar Harbor, ME, USA) were used. Under light-dark (LD) conditions, the Zeitgeber time 0 (ZT0) was designated as lights on and ZT12 was designated as lights off. All animal experiments were conducted in accordance with the guidelines contained in the Act on Welfare and Management of Animals (Ministry of Environment of Japan) and Regulation of Laboratory Animals (Kyushu University) and under the protocols approved by the Institutional Animal Care and Use committee review panels at Kyushu University.

### Intracellular labeling of the cortical microglia in the fixed slice preparations

The wild-type mice were deeply anesthetized with an overdose of sodium pentobarbital (120 mg kg^−1^, i.p.) and perfused transcardially with phosphate-buffered saline (PBS, pH 7.4) followed by 4% paraformaldehyde (PFA) at ZT2 and ZT14. The slices (100 μm thick) were incubated with rabbit anti-Iba1 antibody (1:10,000; Wako Pur Chemical Industies, Ltd., Osaka, Japan, 019–19741) in PBS overnight at 4 °C, followed by incubation with FITC-conjugated donkey anti-rabbit IgG antibody in PBS for 3 h at room temperature. The cells were injected with 4% Lucifer yellow through a glass pipette using a method similar to that previously described^36^,^37^. The sections were then incubated with 4% PFA for 24 h at 4 °C and further stained with an anti-Lucifer yellow antibody (1:50,000, Molecular Probes, A-5750) for 5 days at 4 °C. The section were then visualized with Cy3-conjugated donkey anti-rabbit IgG (1:400, Jackson ImmunoResearch, West Groves, PS, USA) for 3 h at 4 °C.

### Quantitative morphological analyses of the intracellularly labeled cells

All of the images were processed using the ImageJ 1.47 h software program (NIH). Images were obtained with 60 × 1.4 NA oil-immersion lenses and a stack of 30–45 serial optical sections using confocal laser scanning microscope (CLSM, C2si, Nikon, Japan). Microglial processes were traced and then reconstructed as single microglial images using the Simple Neurite Tracer plug-in bundled in FIJI software (freely downloadable from http://fiji.sc/Fiji). These reconstructions were used to calculate the total length of the processes and the branch number as described previously^23^. The morphological skeletonized images of the individual microglia were converted from a binary image using the skeletonize function.

### Two-photon excitation laser scanning microscopy

*CX3CR1*^+/*GFP*^ mice were subjected to two-photon excitation laser scanning microscopy under ketamine and xylazine (100 mg kg^−1^ and 10 mg kg^−1^, respectively) anesthesia. A cranial window (1.8 mm in diameter) was made in the somatosensory cortex, and the dura was carefully removed. *P. gingivalis*, *S. mitis* and *E. coli* were prepared at 3.6 × 10^5^ CFU ml^−1^ in artificial cerebrospinal fluid (ACSF) with 0.5% rhodamine-dextran (Invitrogen, Carlsbad, CA, USA). ATP (10 mM; Sigma-Aldrich Japan K.K., Tokyo, Japan), UDP (10 mM; Sigma-Aldrich), the bacteria mentioned above, *P. gingivalis* LPS (100 μg ml^−1^; Invivogen), and fMLP (10 μM, Sigma Aldrich) were injected into the brain (50–100 μm in depth) through a glass capillary (tip diameter 3–6 μm). PSB0739 (a selective P2Y_12_R inhibitor, 1 μM; Tocris Bioscience, Bristol, UK), MRS2578 (a selective P2Y_6_R inhibitor, 1 μM; Sigma-Aldrich) and apyrase (5 U ml^−1^; Sigma-Aldrich) diluted in ACSF were applied to the surface of the somatosensory cortex for 30 min, and then images were obtained with 16 × 0.8 NA water-immersion lens and a Spectra Physics Mai-Tai IR laser tuned at 920 nm for two-photon excitation of GFP using multiphoton confocal microscope (A1RMP, Nikon, Tokyo, Japan). Z-stack images were taken 1 μm apart from 15 μm above to 15 μm underneath the tip of the capillary every 5 min for 40 min.

### Image quantification

All of the images were processed using the ImageJ 1.47 h software program (NIH). Z-stack images were projected along the z-axis to recreate a two-dimensional (2D) representation of three-dimensional (3D) structures. To quantify the microglial process extension, we measured the number of microglial processes entering area X (within a 40 μm in radius of the glass capillary insertion site) using a method similar to that previously described^25^. To account for any differences in the signal intensity among the experiments, every image was set at the maximum threshold value of all processes (255), and all of the background was set to 0. The number of white pixels in area X over time Rx(t) was measured and compared with the first picture taken immediately after ATP injection Rx(0). The microglial response at any time point R(t) was calculated using the following formula:


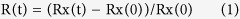


### Microglial cell culture

The c-myc-immortalized mouse microglial cell line, MG6 (RIKEN Cell Bank, Tsukuba, Japan), was maintained in DMEM containing 10% fetal bovine serum (FBS, ICN Biomedicals Japan Co., Tokyo, Japan) supplemented with 10 g ml^−1^ insulin, 100 g ml^−1^ streptomycin, and 100 U ml^−1^ penicillin (BD Falcon, Franklin Lakes, NJ, USA)^38^,^39^. For the infection with various bacteria, MG6 microglial cells were cultured in DMEM without FBS or penicillin.

### Assay for cell survival

MG6 microglial cells (1 × 10^5^ cells ml^−1^) were seeded into 96-well plates (100 μl/well). The cell viability was measured using Cell Counting Kit-8 (Dojin Laboratories, Kumamoto, Japan) under the four different conditions: MG6 microglial cell alone, infected MG6 microglial cells with *P. gingivalis* at multiplicities of infection (MOI) of 1:1, 1:5 and 1:10. At the end of each time point, the medium in the 96-well culture plates was changed to DMEM/F12 to avoid background interference and CCK-8 (10 μl) was added to each well. Absorbance was measured at 450 nm using a microplate reader (620 nm was used as the reference wave length).

### Infection of MG6 microglial cells with bacteria

For the assay of *Il6*, *Tnf*, *Nos2* mRNA expression, MG6 microglial cells were cultured at a density of 1 × 10^6^ cells ml^−1^ in 6-well plates and then infected with *P. gingivalis* at indicated MOI at 37 °C for 3 or 6 h. After incubation, the monolayers were washed three times to remove adherent bacteria and then MG6 microglial cells were collected. In some experiments, MG6 microglial cells were cultured at a density of 5 × 10^5^ cells ml^−1^ in 24-well plates and then infected with *P. gingivalis*, *S. mitis*, or *E. coli* at indicated MOI for 40 min. The supernatants were analysed for the detection of ATP or UDP.

### Isolation of microglia from adult animals

The mice (C57/BL6J, 8–10 weeks old) were anesthetized and perfused transcardially with phosphate-buffered saline (PBS) and then the mice were decapitated (n = 3 at each time point). Microglia were isolated in accordance with the MACS method using magnetically labeled CD11b microbeads, as described previously^23^. The isolated cells were incubated with anti-mouse CD45-fluorescein (FITC) and anti-mouse CD11b-phycoerythrin (PE) at optimal dilutions.

### Quantitative real-time RT-PCR analysis

For examining expression of inflammatory-related molecules by microglia after infection with *P. gingivalis*, total RNA was extracted from MG6 microglial cells at different time points after infection with *P. gingivalis* using RNAiso (Takara Bio Inc., Shiga, Japan). For examining a possible circadian oscillation of the *P2ry6* expression, total RNA was extracted from microglia acutely isolated from the mouse cerebral cortex at different six ZT using the MACS method. For examining expression of *P2ry6* in the somatosensory cortex after administration of P2Y_6_R siRNA, total RNA was extracted from the injection site of the somatosensory cortex.

Complementary DNA (cDNA) was prepared via reverse transcription of the total RNA using a ReverTra Ace qPCR RT kit (Toyobo Co. Ltd., Osaka, Japan). The diluted cDNA samples were analyzed via real-time RT-PCR. Real-time PCR was performed using the THUNDERBIRD SYBR qPCR Mix (Toyobo) and the 7500 Real-time PCR system (Applied Biosystems, Foster City, CA, USA). We determined the copy numbers, via the 2^−ΔΔCt^ method using a calibrator, as previously described^40^. The sequences of primer pairs are described as follows: *Il6*: 5′-TCAATTCCAGAAACCGCTATGA-3′ and 5′-CACCAGCATCAGTCCCA AGA-3′; *Tnf*: 5′-ATGGCCTCCCTCTCAGTTC-3′ and 5′-TTGGTGGTTTGCTAC GACGTG-3′; *Nos2*: 5′-GCCACCAACAATGGCAAC-3′ and 5′-CGTACCGGAT GAGCTGTGAATT-3′; *P2ry6*: 5′-CACTGGCGGACCTGATGTAT-3′ forward and 5′-GGAACAGGATGCTGCCATGTA-3′. For data normalization, an endogenous control (*Actb*).

### Bacterial strains and culture conditions

*P. gingivalis* ATCC33277, *S. mitis* ATCC49456 and *E. coli* DH5α were used. *P. gingivalis* was maintained on blood agar plates and grown in enriched BHI broth (containing, per liter, 37 g of brain heart infusion, 5 g of yeast extract, 1 g of cysteine, 5 mg of hemin, and 1 mg of vitamin K1) under anaerobic conditions (10% CO_2_, 10% H_2_, 80% N_2_)^41^. *S. mitis* and *E. coli* were maintained in enriched BHI and L broth (containing, per liter, 10 g of tryptone, 5 g of yeast extract, and 5 g of sodium chloride) agar plate respectively, at 37 °C in a 5% CO_2_, humidified atmosphere.

### P2Y_6_R knockdown with small interfering RNAs

The P2Y_6_R siRNA (#1: MSS214676, #2: MSS214677, #3: MSS214678, Thermo Fisher Scientific, Waltham, MA, USA), control siRNA (GC duplex negative control), or BLOCK-iT Alexa Fluor Red Fluorescent Control were suspended in Lipofectamine RNAiMAX (Thermo Fisher Scientific). A small craniotomy 0.5 mm in diameter was performed near the imaging area and then the P2Y_6_R siRNA (20 pmol, 5 ml) was directly injected via the craniotomy into the somatosensory cortex of *CX3CR1*^+/*GFP*^ mice 24 h before the *in vivo* imaging analyses.

### ATP and UDP determination

The levels of ATP and UDP in the culture supernatant of *P. gingivalis, S. mitis, E. coli*, and the supernatant of MG6 microglial cells after infection with bacteria were detected using the ATP Determination Kit A22066 (Molecular Probes Inc.) and MicroMolar UDP Assay Kits (ProFoldin Inc., Hudson, MA, USA), respectively, in accordance with the manufacturer’s protocols. The bacterial supernatants were obtained by centrifugation at 6,000× g for 20 min at 4 °C and filter-sterilized using a 0.2 μm filter for the assay. ATP levels were measured by detecting the luminescence using a Berthold Lumat3 LB9508 (Berthold Technologies, Oak Ridge, TN, USA), and the UDP levels were measured by detecting the fluorescence using an EnSight multimode plate reader (PerkinElmer, Waltham, MA, USA).

### Statistical analyses

All of the data are shown as the mean ± SEM. The statistical analyses were performed using a two-tailed unpaired Student’s *t*-test, a one-way analysis of variance (ANOVA) with a post hoc Tukey’s test or a two-way ANOVA with repeated measurements using the GraphPad Prism 7 Software package (GraphPad Software Inc., San Diego, CA, USA). Unless otherwise indicated, the data met the assumptions of equal variances. The differences were considered to be significant at *p* < 0.05.

## Additional Information

**How to cite this article**: Takayama, F. *et al*. Diurnal dynamic behavior of microglia in response to infected bacteria through the UDP-P2Y_6_ receptor system. *Sci. Rep.* **6**, 30006; doi: 10.1038/srep30006 (2016).

## Supplementary Material

Supplementary Videos S1

Supplementary Videos S2

Supplementary Videos S3

Supplementary Videos S4

Supplementary Information
