# Supplementary material for: Diurnal dynamic behavior of microglia in response to infected bacteria through the UDP-P2Y6 receptor system
Source: Sci Rep. 2016 Jul 21;6:30006. doi: 10.1038/srep30006 (PMC4956748; doi:10.1038/srep30006)
Supplement: Supplementary Information [file srep30006-s5.pdf]

SUPPLEMENTARY INFORMATION

for

Diurnal dynamic behavior of microglia in response to infected bacteria through the UDP-P2Y$_6$ receptor system

Fumiko Takayama, Yoshinori Hayashi, Zhou Wu, Yicong Liu, Hiroshi Nakanishi

Supplementary information includes:

Supplementary Figures S1-S5

Supplementary Video S1-S4

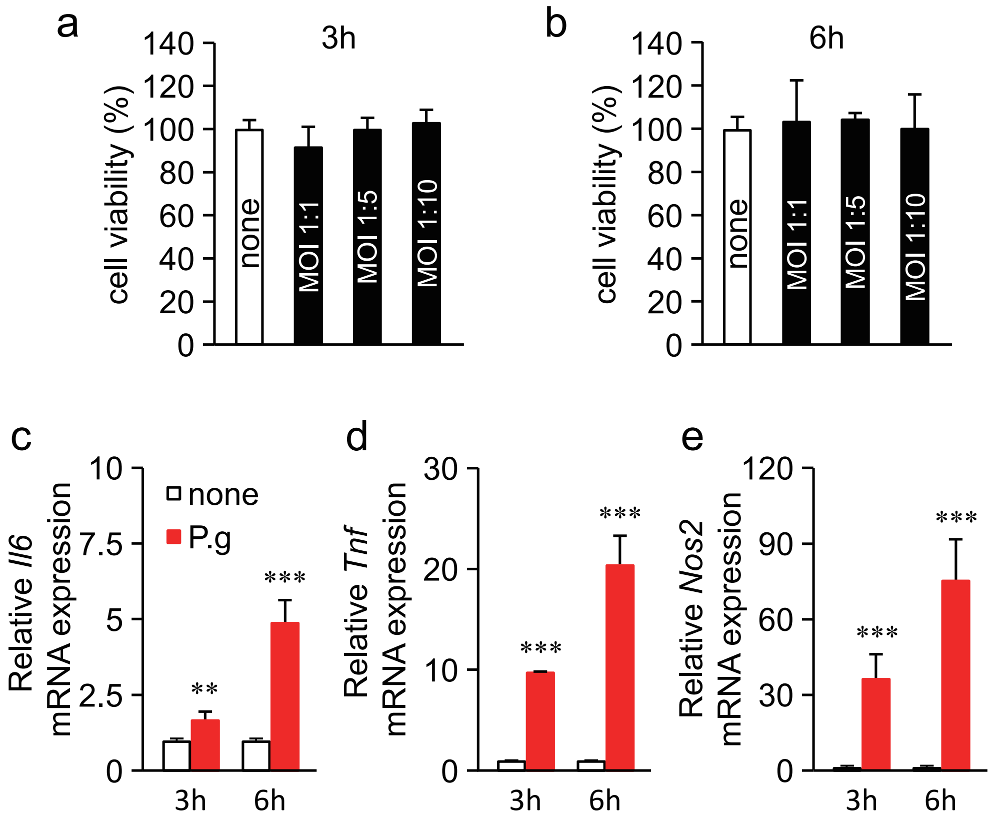

Supplementary Figure S1. Effects on the cell viability and the mRNA expression of proinflammatory-related molecules in MG6 cells following the infection with *P. gingivalis*. (**a,b**) Effects of *P. gingivalis* on cell viability in MG6 after infection with *P. gingivalis* at various MOIs; 1:1, 1:5, 1:10 for 3h (**a**) or 6h (**b**). The data are presented as the mean ± S.E.M. (n = 3, each). A one-way ANOVA with Dunnett's test as compared with non; MOI 1:1, p =0.2911; 1:5, p = 0.9999; 1:10, p = 0.8629 (3h), 1:1, p =0.9767; 1:5, p = 0.9508; 1:10, p = 0.9999 (6h). (**c-e**)The mRNA expression of proinflammatory-related molecules; (**c**) *Il6*, (**d**) *Tnf* (**e**) *Nos2* in MG6 cells after infection with *P. gingivalis* for 3h or 6h at MOI 1:5. The data are presented as the mean ± S.E.M. (n = 3, each). A two-way repeated measure ANOVA with Sidak’ s test (non versus *P. gingivalis*); 3h: **p = 0.0192; 6h: ***p = 0.0001(**c**), 3h: ***p = 0.0001, 6h: ***p = 0.0001 (**d**), 3h: ***p = 0.0001, 6h: ***p = 0.0001 (**e**).

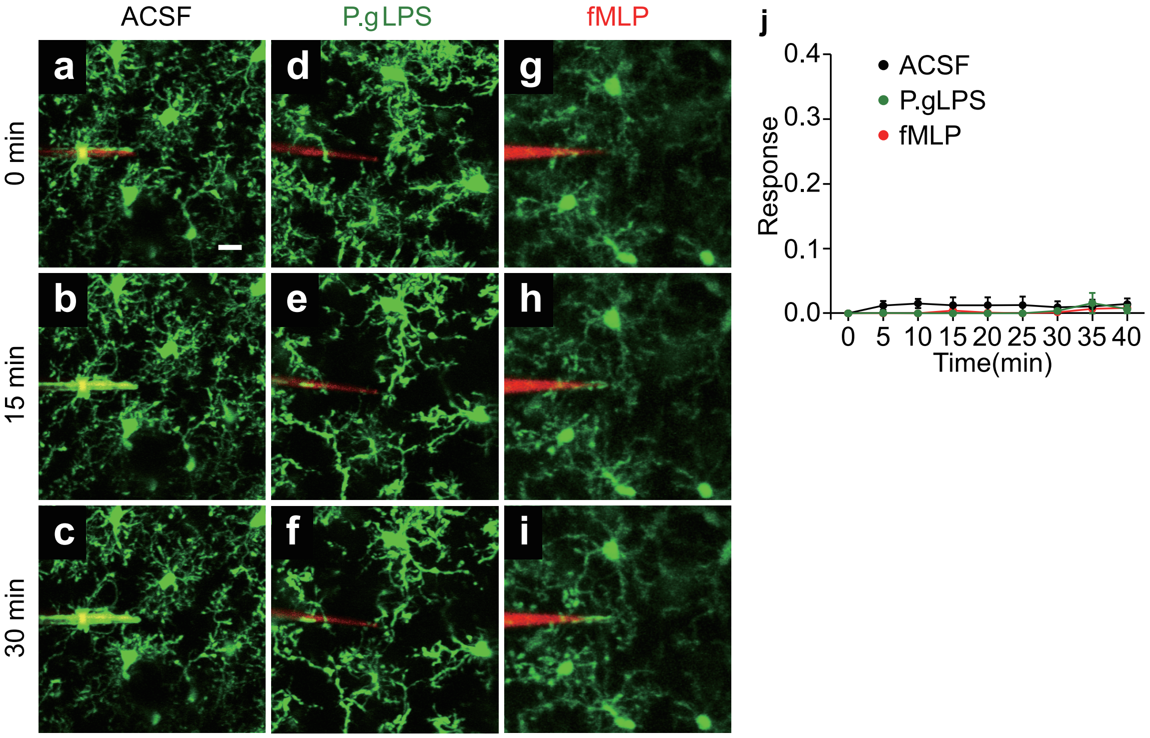

Supplementary Figure S2. *P.g* LPS and fMLP did not induce microglial response to the focal injection area. (**a-c**) ACSF, (**d-f**) 100 μg ml^{-1} *P.g* LPS, (**g-i**) 10 μM fMLP (**j**) The kinetics of the mean fluorescent change of microglial response to ACSF, *P.g* LPS and fMLP. Scale bar: 10 μm. The data are presented as the mean ± S.E.M. (N = 3 mice, n = 3-5 each). Two-way repeated measure ANOVA with Dunnett's test; from 10min to 40min: $p = 0.3078$, $p = 0.4363$, $p = 0.4475$, $p = 0.4212$, $p = 0.8556$, $p = 0.8570$, $p = 0.7224$ for ACSF (control) vs *P. g* LPS, $p = 0.4115$, $p = 0.2542$, $p = 0.6601$, $p = 0.4595$, $p = 0.3636$, $p = 0.6978$, $p = 0.9232$, $p = 0.8096$ for ACSF vs fMLP.

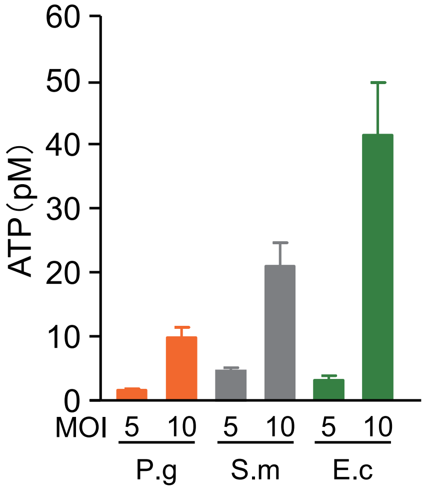

Supplementary Figure S3. MG6 cells released slightly few amount of ATP in response to various bacteria. The data are presented as the mean ± S.E.M. (n = 3 each). MOI 1:5, 1:10, P. g; *P. gingivalis*, S.m; *S. mitis*, E.c; *E. coli*.

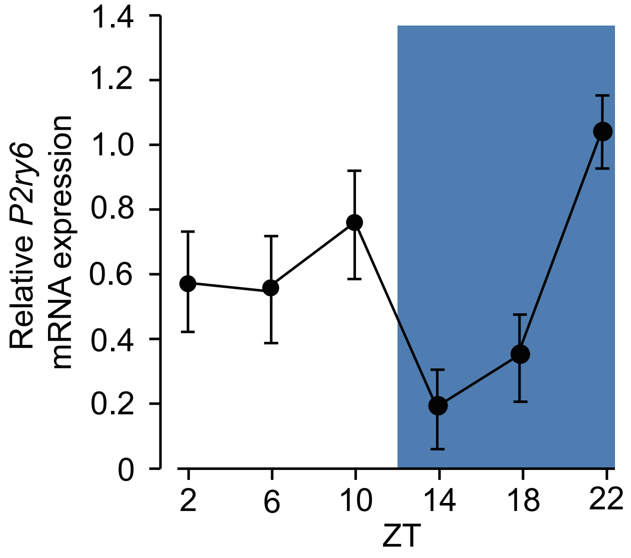

Supplementary Figure S4. Possible associations with the circadian variation of the microglial *P2ry6* expression. The circadian oscillations of the expression of *P2ry6* gene in cortical microglia in mice housed under the LD condition. The data are presented as the mean ± S.E.M. (N=3 mice, n=3 each). One way Repeated measures ANOVA with a post hoc Tukey's test; ZT14 vs ZT10: p =0.0039, ZT14 vs ZT22: p =0.001, ZT22 vs ZT2: p =0.0140, ZT22 vs ZT6: p = 0.0125, ZT22 vs ZT14: p = 0.001, ZT22 vs ZT18: p = 0.0007.

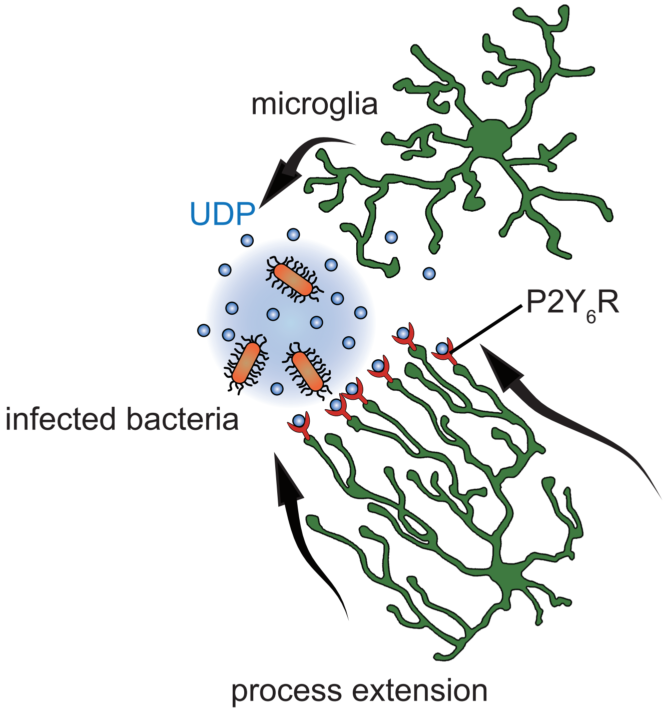

Supplementary Figure S5. A schematic diagram of microglial process extension to the infected bacteria through the UDP-$P2Y_6R$ system. Infected bacteria induce microglial process extension by secretion of UDP from microglia around the infection site .
